# Single-use versus Reusable flexible rhinolaryngoscopes: A retrospective cohort study

**DOI:** 10.1016/j.bjorl.2026.101924

**Published:** 2026-11-04

**Authors:** Yekai Feng, Shisheng Li, Haibo Ou, Qian Yang, Bingbing Zhang, Ou Peng, Ying Zhang

**Affiliations:** Department of Otorhinolaryngology Head and Neck Surgery, The Second Xiangya Hospital, Central South University, Changsha, 410011, Hunan, China

**Keywords:** Single-use flexible rhinolaryngoscope, Reusable flexible rhinolaryngoscope, Operational performance, Safety

## Abstract

**Objectives:**

To describe the real-world clinical application of a single-use flexible rhinolaryngoscope in routine otolaryngology practice.

**Methods:**

This retrospective cohort study included consecutive patients who underwent flexible rhinolaryngoscopy at a tertiary medical center between August 1 and December 1, 2024. Patients were examined using either a Single-use Flexible Rhinolaryngoscope (SFR) or a Reusable Flexible Rhinolaryngoscope (RFR) as part of standard clinical care. All procedures followed institutional protocols. Outcome measures included examination time, treatment time, operator-rated procedural performance (insertion, rotation, withdrawal, and instrument compatibility), subjective image quality, and procedure-related adverse events.

**Results:**

A total of 426 patients were analyzed, including 219 in the SFR group and 207 in the RFR group. Baseline demographic and clinical characteristics were comparable between groups. Mean examination time (SFR: 2.20 ± 1.22 min vs. RFR: 2.18 ± 1.02 min, p = 0.431) and treatment time (SFR: 5.80 ± 3.96 min vs. RFR: 5.35 ± 5.18 min, p = 0.639) did not differ significantly. There was no difference in operator ratings between the two devices regarding insertion, rotation, withdrawal, and compatibility with accessory instruments. Image quality scores were lower for SFR compared with RFR (4.26 ± 0.76 vs. 4.82 ± 0.39, p = 0.001). The SFR was successfully used across a wide range of diagnostic and therapeutic indications, with no device-related adverse events observed.

**Conclusion:**

This single-center retrospective study describes the use of a single-use flexible rhinolaryngoscope in routine otolaryngology practice, with procedures completed without device-related interruptions or adverse events.

**Level of Evidence:**

Level 3.

## Highlights

• Single-use scopes show favorable safety with no device-related adverse events.
• Single-use scopes may reduce cross-contamination and improve workflow.
• Single-use scopes are applicable across a wide range of ENT procedures.

## Introduction

The nasopharynx and larynx are anatomically concealed regions with complex and delicate structures, rendering direct visualization challenging in routine clinical practice. Traditionally, otolaryngologic evaluation of the nasopharynx, hypopharynx, and larynx has relied on indirect mirror examination or rigid laryngoscopy. However, these methods are frequently limited by suboptimal visualization, patient discomfort, and insufficient exposure, particularly in individuals with an exaggerated gag reflex, anxiety, narrow nasopharyngeal anatomy, or a prominent epiglottis.^1^^,^^2^

Flexible fiberoptic rhinolaryngoscopes enables direct visualization of pathological lesions and has become a cornerstone of modern otolaryngologic practice.^3^ Nevertheless, image quality is intrinsically constrained by the number and resolution of optical fibers. In contrast, electronic flexible rhinolaryngoscopes provide a wider field of view and high-resolution dynamic imaging, thereby improving diagnostic accuracy and facilitating therapeutic interventions. Despite these advantages, their clinical application is limited by substantial maintenance and reprocessing costs, prolonged turnaround times, and the persistent risk of cross-contamination due to exposure to blood, bodily fluids, and microorganisms during use.^4^^,^^5^ During the Coronavirus 2019 pandemic, ﬂexible endoscopy of the upper airway was identiﬁed as a potentially highly contagious procedure.^6^ Moreover, inadequate endoscope reprocessing and deviations from established reprocessing guidelines have been frequently reported, further amplifying concerns regarding infection control.^7^

Several strategies, including the use of disposable protective sheaths, have been proposed to mitigate infection risk and simplify reprocessing workflows; however, these measures do not completely eliminate the potential for contamination and device-related infection.^8^ Compared with Reusable Flexible Rhinolaryngoscope (RFR), Single-use Flexible Rhinolaryngoscope (SFR) integrate the imaging advantages of electronic nasopharyngoscopes with the inherent benefits of disposability. By eliminating the need for reprocessing, SFR reduce the risk of cross-infection while offering operational convenience. Prior studies in related fields have demonstrated that single-use flexible bronchoscopes are noninferior to reusable devices with respect to procedural performance, while providing superior infection control and workflow efficiency.^9^^,^^10^ These characteristics make single-use devices particularly attractive for patients at high risk of infection and for use in emergency or high-throughput clinical settings.^5^

Although the number of studies comparing single-use and reusable optical devices has increased substantially in recent years, particularly in gastrointestinal endoscopy, bronchoscopy, and urology,^9^^,^^11^^,^^12^ research specifically evaluating SFR versus RFR in real-world otolaryngology clinical settings remains very limited. Therefore, the present study aimed to describe the clinical use of a single-use flexible rhinolaryngoscope in routine practice based on real-world data.

## Methods

### Subjects and study design

This retrospective cohort study was approved by the Clinical Research Ethics Committee of The Second Xiangya Hospital of Central South University (approval number: 02220206). Written informed consent was obtained from all individual participants or their legal guardians prior to their inclusion in the study. Prior to the start of this study, both types of rhinolaryngoscope had been routinely used in the ENT department for a period of time. All procedures were performed by three board-certified otolaryngologists who had received formal training in rhinolaryngoscopy. Each operator had independently completed a minimum of five procedures with both SFR and RFR prior to patient enrollment, ensuring familiarity with device handling and minimizing operator-related variability.

Consecutive adult outpatients who underwent examination and/or treatment using either a SFR or a RFR between August 1 and December 1, 2024, were included. Patients were allocated to either the SFR or RFR group according to endoscope availability and clinical scheduling on the day of the procedure. Throughout the 4-month study period, the two endoscope types were alternately assigned on a weekly basis, with each type being used for eight consecutive weeks. During weeks designated for SFR use, all eligible patients underwent diagnostic or therapeutic procedures with the SFR, whereas during weeks designated for RFR use, all eligible patients were examined with the RFR. Relevant clinical data and safety outcomes were recorded prospectively in real time during the procedures, and follow-up continued until patient discharge. Inclusion criteria were as follows: 1) Age ≥ 18-years; 2) Patients who underwent diagnostic or therapeutic rhinolaryngoscopy using SFR or RFR during routine clinical care.

### Equipment

Patients in this study were examined and treated using either a single-use flexible rhinolaryngoscope (Vathin E-SteriScope™ RL-S1800/RL-S1E00; Olympus Corporation) or a reusable flexible rhinolaryngoscope (ENF-V3/ENF-VT3; Olympus Corporation). The principal technical specifications of the four rhinolaryngoscope models are summarized in Table 1. Representative photographs of the SFR and RFR devices are provided in Fig. 1.

**Table 1:** Features of RFR and SFR.

Equipment parameters	RFR		SFR	
	ENF-V3	ENF-VT3	RL-S1800	RL-S1E00
Insertion Tube OD (mm)	2.6	4.8	3.2	4.9
Distal and OD (mm)	2.9	4.9	3.2	5
Channel ID (mm)	0	2	0	2.2
Bending (up/down)	130°/130°		210°/210°	
Rotary Function (left/right)	70°		90° ± 10%	
Working Length (mm)	300	365	300	
Depth of Field (mm)	3.5‒50	2‒50	3‒100	
Field of View	90		110° ± 15%	
Risk of cross infection	Yes		No	
Portability	No		Yes	

OD, Outer Diameter; ID, Inner Diameter.

**Fig. 1:**
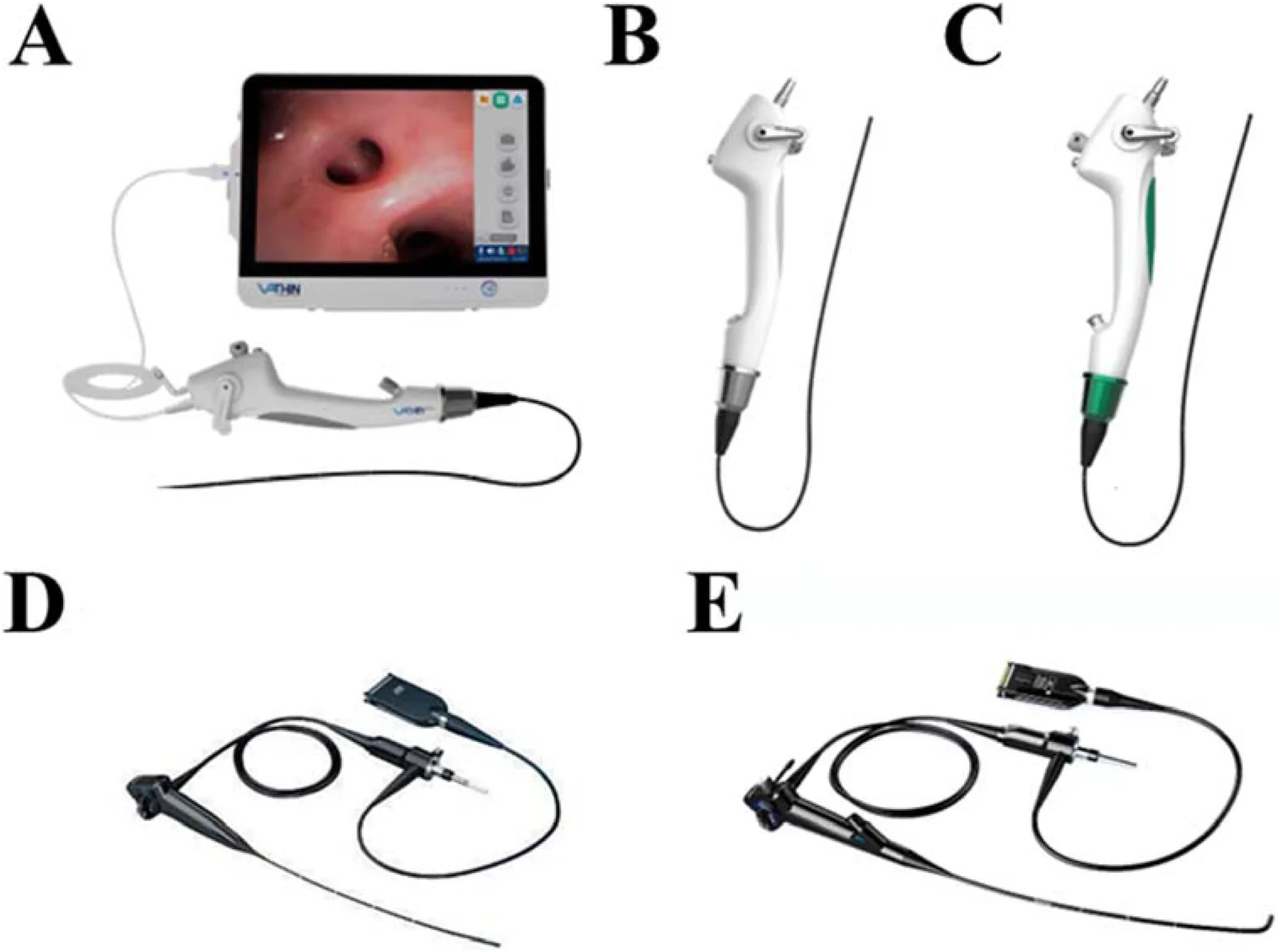
Representative photographs of the SFR and RFR devices. (A) The portable console unit with an attached SFR. (B–C) Two models of the SFR: RL-S1800 (B) and RL-S1E00 (C). (D–E) Two models of the RFR: ENF-V3 (D) and ENF-VT3 (E).

## Operation methods

### Routine rhinolaryngoscopic examination and intervention

Rhinolaryngoscopy was performed under topical surface anesthesia or local anesthesia according to routine clinical practice. The rhinolaryngoscope was introduced transnasally and advanced with controlled bending and rotation to obtain systematic visualization and image acquisition of the nasopharynx, hypopharynx, and larynx, followed by device withdrawal. Mucosal color, surface characteristics, and subtle pathological changes were carefully evaluated, and therapeutic interventions were performed when indicated. The operators recorded objective data, including the start and end times of the examination, any medical issues encountered, and the completion status of the procedure.

### Performance evaluation

The evaluation was conducted in real time by the operators based on their observations during the procedures. Evaluation domains encompassed insertion, rotation, withdrawal, image quality, and compatibility with accessory instruments during both diagnostic and therapeutic procedures. We followed previously published studies comparing single-use and reusable endoscopes and employed a 5-point Likert scale, where 1 indicated very poor, 2 poor, 3 acceptable, 4 good, and 5 excellent. This approach has been widely validated in medical research and demonstrates strong reliability and validity.^9^^,^^13^, ^14^, ^15^

### Adverse event assessment

Safety endpoints included procedure-related adverse events occurring during or immediately after rhinolaryngoscopy. Monitored events comprised patient discomfort, sneezing, coughing, nausea or vomiting, laryngospasm, nerve injury, transient elevation of blood pressure, and hemorrhage.

### Statistical considerations

Statistical analyses were performed using SPSS software (IBM Corp., Armonk, NY, USA). Continuous variables were summarized as mean ± standard deviation, while categorical variables were expressed as counts and percentages. Comparisons between groups were conducted using the Chi-Square (χ²) test for categorical variables. For continuous variables, between-group comparisons were performed using the independent-samples *t*-test, and one-way analysis of variance (ANOVA) was applied when comparisons involved more than two groups. A two-sided p-value < 0.05 was considered statistically significant.

## Results

### Patients’ clinical characteristics

A total of 426 eligible patients were included in the analysis. Of these, 219 patients underwent procedures using the SFR, including 113 diagnostic examinations and 106 therapeutic procedures. The RFR group comprised 207 patients, including 109 diagnostic examinations and 98 therapeutic procedures.

The overall cohort had a mean age of 52-years (range 18–96 years). Baseline demographic and clinical characteristics were comparable between the two groups and are summarized in Table 2. The primary indications for rhinolaryngoscopy included pharyngitis, laryngopharyngeal reflux, laryngeal masses, malignant tumors of the larynx, and obstructive sleep apnea-hypopnea syndrome (snoring). A detailed distribution of diagnoses is presented in Table 3.

**Table 2:** Participant demographics.

Characteristic	RFR		SFR		p-value^a^
	Examination (n = 109)	Treatment (n = 98)	Examination (n = 113)	Treatment (n = 106)	
Age (years, mean ± SD)	53.7 ± 14.7	53.9 ± 14.4	51.0 ± 12.4	52.4 ± 14.2	0.37
BMI, (kg/m^2^, mean ± SD)	23.2 ± 3.5	23.6 ± 3.3	23.5 ± 3.6	23.1 ± 2.8	0.54
Gender					0.41
Male, n (%)	60 (45%)	59 (60.2%)	56 (49.6%)	67 (63.2%)	
Female, n (%)	49 (55%)	39 (39.8%)	57(50.4%)	39 (36.8%)	
Deviated nasal septum					0.06
Yes, n (%)	37 (33.9%)	15 (13.5%)	52 (46%)	11 (10.4%)	
No, n (%)	72 (66.1%)	83 (84.7%)	61 (54%)	95 (86.9%)	

a p-values were calculated using *χ*^2^ tests for categorical variables and One-Way ANOVA for continuous variables. A p-value of 0.05 was considered significant.

**Table 3:** Preoperative diagnosis of enrolled patients^a^.

Diagnosis, n (%)	RFR (n = 207)	SFR (n = 219)
Epistaxis	NA	1 (0.45%)
Nasopharyngeal examination	NA	1 (0.45%)
Pharyngitis	56 (27.05%)	58 (27.62%)
laryngopharyngeal reflux	7 (3.38%)	5 (2.28%)
Foreign body in pharynx	37 (17.87%)	29 (13.24%)
Status post pharyngeal surgery	4 (1.93%)	5 (2.28%)
Benign tumors in parapharyngeal space	1 (0.48%)	6 (2.74%)
Pharyngeal mass	27 (13.04%)	32 (14.61%)
Malignant tumours of larynx	18 (8.70%)	21 (9.59%)
Laryngitis	3 (1.45%)	1 (0.45%)
Tonsil hypertrophy	4 (1.93%)	3 (1.37%)
Chronic tonsillitis	5 (2.42%)	3 (1.37%)
Postoperative epiglottic cyst	2 (0.97%)	4 (1.83%)
Vocal cord polyp	6 (2.90%)	8 (3.65%)
Vocal cord paralysis	4 (1.93%)	2 (0.91%)
Vocal cord congestion	1 (0.48%)	2 (0.91%)
Vocal leukoplakia	6 (2.90%)	4 (1.83%)
Thyroid malignancy	2 (0.97%)	3 (1.37%)
Upper airway resistance syndrome	1 (0.48%)	2 (0.91%)
Obstructive sleep apnea-hypopnea syndrome (snoring)	17 (8.21%)	20 (9.13%)
Dysacusis	1 (0.48%)	1 (0.45%)
Cough	NA	1 (0.45%)
Tinnitus	1 (0.48%)	1 (0.45%)
Hoarseness	2 (0.97%)	2 (0.91%)
Neck abscess	1 (0.48%)	1 (0.45%)
Localized neck swelling	1 (0.48%)	3 (1.37%)

a The preoperative diagnosis was established through preoperative examinations conducted prior to Rhinolaryngoscope, encompassing visual inspection and traditional laryngoscopy examination.

### Examination and treatment time

The mean examination time did not differ significantly between groups. In the RFR group, the mean examination time was 2.18 ± 1.02 min (95% CI 1.99–2.38), compared with 2.20 ± 1.22 min (95% CI 1.99–2.44) in the SFR group (p = 0.431) (Fig. 2A). Similarly, the mean treatment time was 5.35 ± 5.18 min (95% CI 4.37–6.34) for the RFR group and 5.80 ± 3.96 min (95% CI 5.13–6.70) for the SFR group (p = 0.639) (Fig. 2B). Most diagnostic examinations were completed within 3-minutes: 97 cases (88.9%) in the RFR group and 108 cases (95.5%) in the SFR group. As expected, therapeutic procedures required more time. Among treatment procedures, 74 cases (75.5%) in the RFR group and 73-cases (68.9%) in the SFR group were completed within 6-minutes. No patients required repeat procedures due to incomplete examination or treatment.

**Fig. 2:**
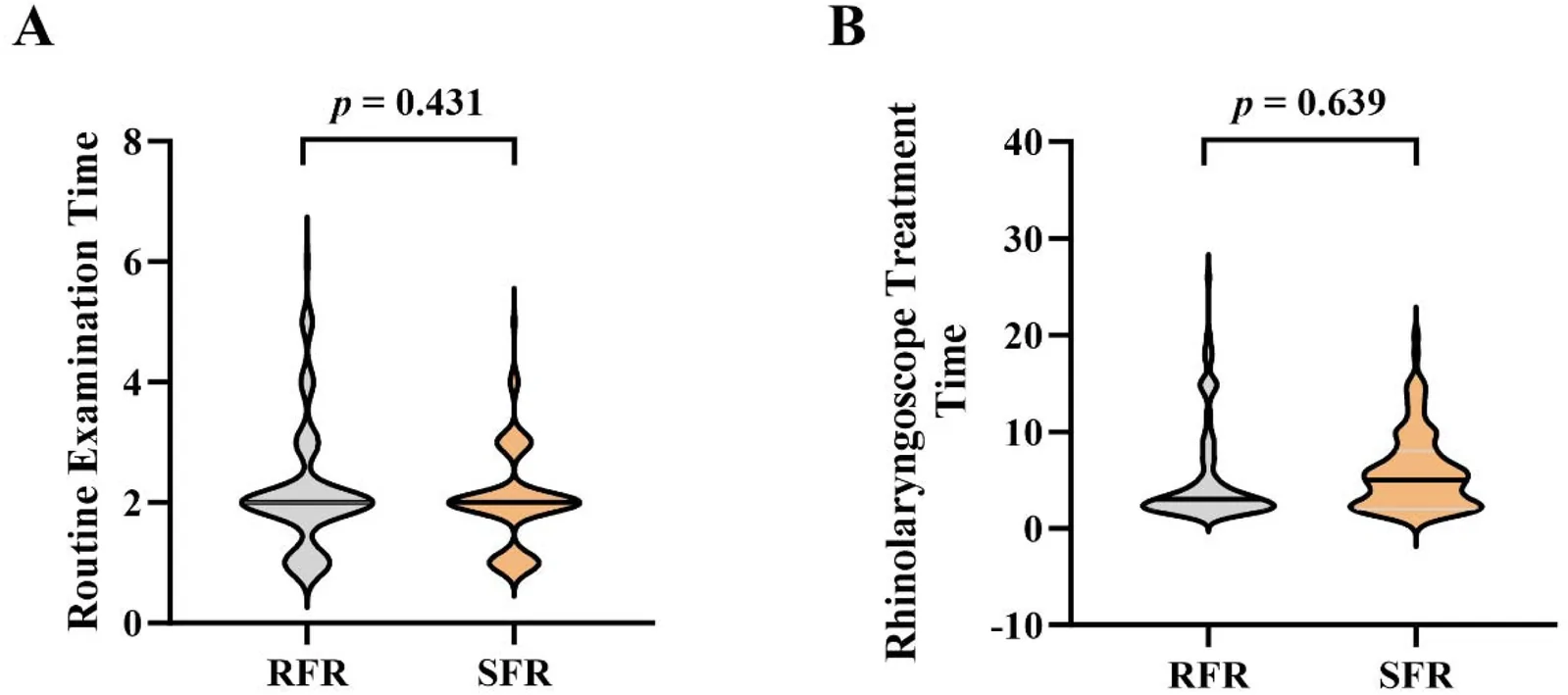
(A) Comparison of Routine Examination Time between RFR vs. SFR. (B) Rhinolaryngoscope treatment time from RFR vs. SFR.

### Assessment of operational performance

The operational performance of the rhinolaryngoscopes was evaluated by three experienced operators (Table 4). Among the 219 patients examined with the SFR, all procedures were completed successfully except for one case of leakage due to rapid injection of fluid and one case of unsuccessful foreign body removal. Operators noted that the SFR, with its portable console, offers significant portability, enabling its use across diverse clinical settings such as inpatient wards, outpatient/emergency departments, and the ICU. For procedural performance, the SFR demonstrated comparable results to the RFR in insertion, rotation, withdrawal, and compatibility with other surgical instruments. However, image quality was slightly lower for the SFR (4.26 ± 0.76) compared with the RFR (4.82 ± 0.39). In this study, the SFR group exhibited a higher standard deviation compared with the RFR group. We further analyzed the distribution of image quality scores across groups (Table 5): in the SFR group, 19.6% of patients scored 3, 35.2% scored 4, and 45.2% scored 5, whereas in the RFR group, 0.5% scored 3, 18.4% scored 4, and 81.1% scored 5. Representative images illustrating the differences in image quality between RFR and SFR are shown in Fig. 3. 1A and 2A display normal hypopharyngeal anatomy captured by RFR and SFR, respectively. 1B–C and 2B–C show various laryngeal lesions, including tumors and polyps, imaged with RFR and SFR. Therefore, despite a slightly lower image quality score, the SFR still provides clear visualization of pathological lesions.

**Table 4:** Performance evaluation scores of SFR and RFR.

Access of operation	RFR	SFR	p-value^a^
Insertion (mean ± SD)	4.68 ± 0.48	4.60 ± 0.52	0.11
rotation (mean ± SD)	4.86 ± 0.35	4.79 ± 0.41	0.08
withdrawal (mean ± SD)	4.86 ± 0.62	4.84 ± 0.38	0.57
image quality (mean ± SD)	4.82 ± 0.39	4.26 ± 0.76	0.001
compatibility with accessory instruments (mean ± SD)^b^	4.72 ± 0.47	4.73 ± 0.51	0.98

a Student’s *t*-test (p < 0.05) for continuous variables.

b Device compatibility assessed during treatment in SFR and RFR groups.

**Table 5:** Rating of image quality during the procedure.

Category	Number of patients (%)	
	RFR	SFR
Very poor	0 (%)	0 (%)
Poor	0 (%)	0 (%)
Acceptable	1 (0.5%)	43 (19.6%)
Good	38 (18.4%)	77 (35.2%)
Excellent	168 (81.1%)	99 (45.2%)

**Fig. 3:**
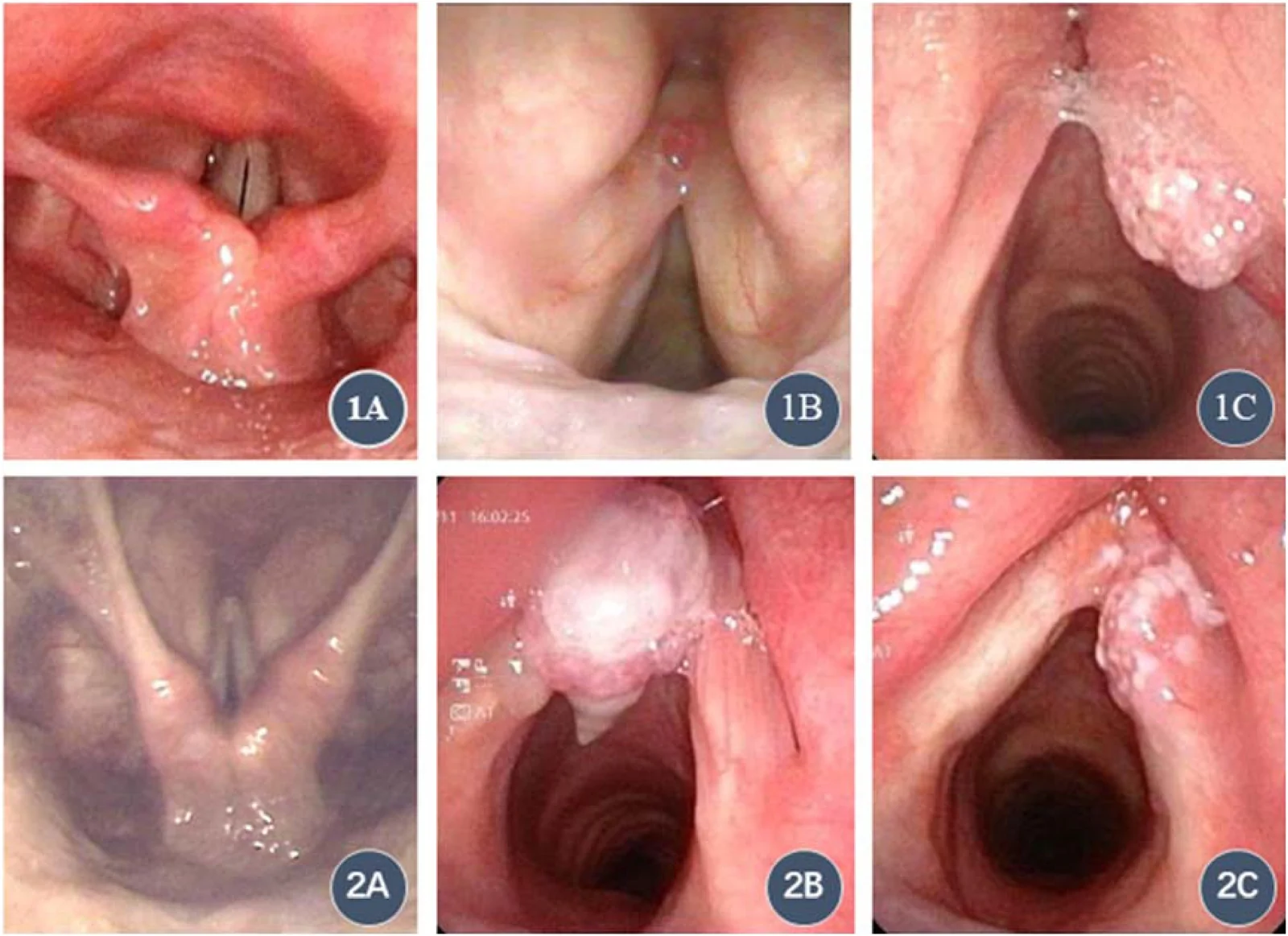
Comparison of imaging changes between RFR and SFR. (A–C) Representative images of RFR. (A–C) Representative images of SFR.

### Safety assessment of the SFR

The safety of the SFR was evaluated based on changes in patients’ signs and symptoms during and immediately after the procedures. Among patients undergoing diagnostic procedures, seven in the SFR group and eight in the RFR group reported mild postoperative discomfort. Symptoms were minor, did not interfere with the procedure, and did not require any specific intervention. One patient in the SFR treatment subgroup developed a transient cough following a successful procedure, which also required no intervention. No other adverse events were observed. No patient developed an infection following endoscopic examination and treatment.

### SFR application scenarios

A total of 219 patients were included in the SFR group, encompassing six primary clinical scenarios (Table 6): 1) For patients presenting with symptoms such as nasal obstruction or rhinorrhea, where nasoendoscopy provided superior visualization and enabled biopsy for definitive diagnosis; 2) For patients with suspected or confirmed foreign bodies, where endoscopy was used for direct visualization and to guide therapeutic removal; 3) For patients with identified lesions requiring histopathological confirmation via directed vocal cord or laryngeal biopsy; 4) For patients in whom conventional indirect laryngoscopy failed due to anatomical constraints (e.g., prominent gag reflex), necessitating a transnasal endoscopic approach; 5) For patients undergoing diagnostic evaluation to detect occult laryngeal pathology, screen for early-stage tumors, or assess vocal cord mobility; 6) For patients with small vocal cord lesions (e.g., polyps, nodules) who underwent endoscopic resection under direct visualization.

**Table 6:** Indications covered by SFR group (n = 219).

Primary Indications	Examination (n)	Treatment (n)	Total (n)
Unexplained bleeding, inflammation, or lesions in the nasal cavity/nasopharynx/pharynx.	61	Biopsies: 20	81
Localization and assessment of foreign bodies within the nasal cavity or pharynx.	2	Foreign body removal: 27	29
Acquisition of tissue biopsies or cytologic specimens for definitive pathological diagnosis.	NA	Biopsies: 48	48
Examination in cases where indirect laryngoscopy is precluded (e.g., due to severe gag reflex, prominent dentition, or an elevated tongue base).	37	NA	37
Detection of subtle laryngeal lesions or early-stage tumors, with evaluation of vocal cord mobility.	13	Biopsies: 2; Cricoarytenoid joint reduction：1	16
Surgery for small vocal cord lesions (e.g., polyps, nodules).	NA	Vocal cord surgery: 8	8

## Discussion

Electronic flexible nasopharyngolaryngoscopes provide high-resolution imaging and intuitive operation, enabling clear visualization of complex anatomical structures within the ear, nose, and throat. Consequently, these devices have been widely adopted in clinical practice. Reusable electronic nasopharyngoscopes are currently the standard in most clinical settings, but their use requires substantial time and resources for cleaning and prevention of cross-contamination.^16^ In contrast, clinical evidence regarding the use of single-use electronic nasopharyngoscopes remains limited. Gudnadottir et al. conducted a study comparing Ambu® single-use nasopharyngoscopes with Karl Storz reusable nasopharyngoscopes, but only included 26 procedures and did not involve therapeutic interventions such as biopsies, foreign body removal, or vocal cord procedures.^15^ Kidane et al. assessed the environmental impact of single-use versus reusable laryngoscopes from a lifecycle perspective.^17^ In this study, we analyzed clinical data from 426 patients who underwent examination or treatment with either the RFR or SFR, allowing a real-world comparison of their clinical application in routine otolaryngology practice.

There were no significant differences in examination or treatment times between the SFR and RFR devices. The SFR performed effectively across a range of procedures, including routine rhinolaryngoscopic examinations, foreign body removal, targeted biopsies, and vocal cord interventions. Notably, a high proportion of procedures were completed within practical timeframes (e.g., over 88% of diagnostic examinations were completed within 3-minutes), and no patients required repeat procedures, highlighting the reliability and procedure completion of the SFR. The incidence of intraoperative and postoperative adverse events was extremely low. Only a few patients reported mild postoperative discomfort or transient coughing, none of which required medical intervention. No serious complications, such as laryngospasm or hemorrhage, were observed.

At our study center, all RFRs were used strictly in accordance with standardized endoscope reprocessing protocols,^16^ and no infections were observed among patients in the RFR group during the study period. Nevertheless, prior literature indicates that reusable endoscopes carry an inherent infection risk. For example, during the COVID-19 pandemic, a study reported that among 8337 outpatients undergoing laryngoscopy, one patient tested positive for SARS-CoV-2 within 10-days post-procedure, despite no healthcare personnel being infected.^18^ Other reports have documented hepatitis B and C transmission during colonoscopy.^19^ Studies have also shown that reusable flexible bronchoscopes can lead to cross-contamination,^20^^,^^21^ with infection rates of up to 2.8%.^20^ Thus, while RFR are generally safe under strict reprocessing, SFR offer additional protection in high-risk settings, particularly for immunocompromised patients or when minimizing nosocomial cross-infection is critical. Using SFR not only helps protect healthcare personnel but also reduces potential infection risk to patients, providing a higher level of safety in clinical practice.

Evaluation of operational performance revealed that the SFR was rated favorably and was equivalent to the RFR across most ergonomic and functional domains, including insertion, rotation, withdrawal, and compatibility with accessory instruments. These findings are consistent with prior studies on single-use endoscopes.^22^^,^^23^ The comparable handling characteristics suggest that clinicians can adopt the SFR without a significant learning curve or compromise in procedural performance. Image quality assessment showed that the SFR had lower scores and a higher standard deviation than the RFR, indicating that the overall consistency of image quality was lower for the SFR. This suggests that the imaging stability of the SFR could be further enhanced in future iterations.

The study population encompassed a wide spectrum of common ENT conditions, confirming the applicability of the SFR across six key clinical scenarios, from routine diagnostic evaluation to foreign body removal and directed biopsies. The SFR effectively eliminates the risk of cross-contamination associated with inadequate reprocessing of reusable scopes. Furthermore, these devices significantly shorten turnover time and enhance procedural efficiency. Coupled with their notable portability, this profile provides a clear advantage in managing patients at high risk of infection. The ease of moving the equipment renders them particularly suitable for conducting assessments in diverse clinical environments such as wards, the emergency department, and the intensive care units.

### Limitations

This study has several limitations. First, the findings are based on specific models of single-use and reusable flexible rhinolaryngoscope from a single manufacturer, and therefore cannot be directly generalized to other models or brands, as design differences between manufacturers may affect image quality and operational performance. Second, a formal sample size calculation was not performed due to the retrospective design and real-world consecutive case inclusion. Third, differences in appearance, interface, and handling between the SFR and RFR precluded blinding of the operators during procedures. To mitigate familiarity bias, both devices had been routinely used in the department prior to study initiation, and all operators had completed at least five procedures with each device. Nevertheless, operators inevitably had greater overall experience with the RFR.

In the real-world clinical setting, inherent limitations of the devices’ data interfaces prevented acquisition of directly comparable quantitative parameters, such as resolution, contrast, and signal-to-noise ratio, making simultaneous objective assessment of image quality during procedures unfeasible. Future studies should consider multi-center, multi-manufacturer designs and integrate objective physical measurements with standardized subjective scoring to achieve a more comprehensive and generalizable evaluation of device performance.

Finally, this study focused on clinical applicability and did not include a cost-analysis comparison between SFR and RFR. Future studies should incorporate comprehensive health economic evaluations, including local device acquisition costs, reprocessing costs, labor and time expenditures, device lifespan, waste management fees, and potential infection control–related expenses, in order to systematically compare the long-term cost-effectiveness of SFR and RFR across different healthcare settings.^23^

## Conclusion

This single-center retrospective study describes the clinical use of a single-use flexible rhinolaryngoscope in routine otolaryngology practice, with procedures completed as planned without device-related technical difficulties or adverse events. Further prospective studies with larger sample sizes are warranted to validate these observations.

## ORCID ID

Yekai Feng: 0000-0002-8069-7009

Shisheng Li: 0000-0001-5874-6049

Haibo Ou: 0009-0002-2529-6824

Qian Yang: 0000-0002-9939-5131

Bingbing Zhang: 0009-0003-9590-5987

Ou Peng: 0009-0000-3962-7896

## Ethics approval and consent to participate

This study was approved by the the Ethics Committee of The Second Xiangya Hospital of Central South University (approval number: 02220206). All methods were performed in accordance with the Declaration of Helsinki, and informed consent was obtained from all participants.

## Data availability

Data can be made available via specific request to the Corresponding Author.

## Declaration of competing interest

The authors declare that the research was conducted in the absence of any commercial or financial relationships that could be construed as a potential conflict of interest.
